# Evaluation of the Insecticidal Activities of α-Pinene and 3-Carene on *Sitophilus zeamais* Motschulsky (Coleoptera: Curculionidae)

**DOI:** 10.3390/insects11080540

**Published:** 2020-08-17

**Authors:** Jacob D. Langsi, Elias N. Nukenine, Kary M. Oumarou, Hamadou Moktar, Charles N. Fokunang, George N. Mbata

**Affiliations:** 1 Faculty of Sciences, University of Ngaoundere, Ngaoundere P. O. Box 454, Cameroon; langsijacob@gmail.com (J.D.L.); ennukenine@fulbrightmail.com (E.N.N.); Hamadoumoktar0@gmail.com (H.M.); 2 National School of Veterinary Medicine and Sciences (ESMV), Ngaoundere P. O. Box 454, Cameroon; Oumarou_mallamkary92@yahoo.fr; 3 Faculty of Medicine and Biomedical Sciences, University of Yaoundé 1, Yaounde P. O. Box 33032, Cameroon; charlesfokunang@yahoo.co.uk; 4 Agriculture Research Station, Fort Valley State University, 1005 State University Drive, Fort Valley, GA 31030, USA

**Keywords:** monoterpenes, stored product protection, fumigation, repellence, maize weevil

## Abstract

**Simple Summary:**

In order to assure food security, proven postharvest pest management tools are needed to secure grains and processed food from insect infestations. Plants and plant products have over the years been proven to possess great insecticidal properties. Farmers in Cameroon use cypress leaves (*Cupressus sempervirens*) as pesticide against the maize weevil (*Sitophilus zeamais*). Two pure compounds (α-Pinene and 3-Carene) from cypress were tested against the maize weevil for contact and fumigant toxicities. Both compounds demonstrated efficacies against both adult and immature weevils. These compounds did not change the color of the treated stored maize and could be exploited as novel maize insecticides.

**Abstract:**

Pest management in most sub-Saharan subsistence agriculture involves mainly the use of botanicals that are either applied as powders, solvent extracts, ash or essential oils. Two hydrogenated monoterpenes (α-pinene and 3-carene) from *Cupressus sempervirens* were tested against *Sitophilus zeamais* in the laboratory to evaluate the contact and fumigation effects on the mortality of adult and immature weevils, progeny production, and grain damage. Contact toxicity of the terpenes was investigated at these concentrations: 0.08, 4, 8 and 12 ppm (terpene/maize), while fumigant action was studied at the following doses: 1, 2, 3, and 4 ppm. The results indicate that insecticidal effects were concentration-dependent since mortality increased with dosage and exposure periods. After a 14-day exposure period at the concentration of 12 ppm of α-pinene and 3-carene/grain, more than 98% mortality of the mature weevils was observed at concentrations of 4.1333 and 1.642 ppm respectively and progeny production was reduced by 98% and 100%, respectively. When α-pinene and 3-carene were applied as fumigants, LC_50s_ (lethal concentrations that generate 50% mortality) of 1.402 and 0.610 ppm were obtained after 24 h of exposure, respectively. At concentrations above 3 ppm, both monoterpenes acted as repellents to weevils and reduced grain damage by 80%. Both monoterpenes inhibited the development of immature stages of the weevil and reduced progeny by up to 94%. These compounds are very promising and effective and could be exploited as novel phytoinsecticides against the maize weevil.

## 1. Introduction 

Sustainability of food security is not solely dependent on intensive crop cultivation. Successful storage of harvested produce is key to guarantee food availability at all times. In Cameroon, maize (*Zea mays* L.) is a staple and its cultivation is mainly by small-scale subsistence farmers. Maize is a source of food for both humans and livestock [1,2]. *Sitophilus zeamais* Motschulsky (Coleoptera: Curculionidae) is the most important postharvest pest of maize and is capable of causing losses greater than 80% of unprotected maize in 6 months [3,4]. Pest management in some storage pests could be very challenging with immature stages of insect pests that develop internally in grains and are therefore more tolerant to pesticides [5,6]. Generally, management of the maize weevil involves the use of synthetic pesticides, botanicals as well as entomopathogenic agents [6,7]. In Cameroon, in addition to wrong usage of chemical pesticides, the tools for pest management are mainly botanicals. These botanicals are either applied as powders, solvent extracts, ashes or essential oils [3,6]. Essential oils have been shown to be very complex and diversified in phytochemical composition [8,9]. The variability in chemical composition of essential oils is the basis for their activity [7,9]. GC-MS analysis usually shows that essential oils are mainly composed of different monoterpene classes (oxygenated or hydrogenated mono-, di-, tri- or even sesqui-terpenes [10,11]. This diversity of monoterpene compounds has given essential oils the ability to possess a wide range of pesticidal activity against different postharvest pests and microbes [7,12,13,14,15]. Monoterpenoids for instance have been demonstrated to have varying degrees bioactivity against different storage pests [6,7,14,15,16,17,18]. There is overwhelming presence of hydrogenated monoterpenes in essential oils of *Cupressus sempervirens* and *Chenopodium ambrosioides* [8,10]. These two essential oils contain 69.2% and 79.87% of hydrogenated monoterpenes, respectively. In *C. sempervirens*, 3-carene makes up 25.91% while α-pinene makes up 17.59%. The goal of this study was to evaluate the bio-efficacies of both hydrogenated monoterpenes against *S. zeamais* by contact and fumigation applications by determining their effects on weevil mortality, progeny production, repellence, development of immature stages, and reduction of grain damage by the weevil.

## 2. Materials and Methods

### 2.1. Insects and Maize Substrate

The Acid-Tolerant Population (ATP) variety of maize was collected from IRAD (Institute of Agricultural Research for Development) Wakwa (Ngaoundere). The maize was sorted, washed, sun-dried and disinfested by storing them in a freezer at −13 °C for two weeks. Afterwards, they were removed and left on laboratory shelves for acclimatization. The maize moisture content was determined to be 12.591% by the oven method as described in an earlier study [19]. 

Adult weevils were obtained from the Laboratory of Applied Zoology, University of Ngaoundere, Cameroon. These insects were maintained in culture in 5 L transparent glass jars containing 2 kg of previously disinfested maize on laboratory shelves at a temperature range of 17.3–28.8 °C and 56.3–97.8% RH. 

### 2.2. Terpenes Used in the Study—A-Pinene and 3-Carene 

Pure α-pinene and 3-carene from Thermo Fisher Scientific (One Reagent Lane, Fair Lawn, NJ 07410, USA) were used for the tests. All tests were carried out from January to June 2020 in the Laboratory of Applied Zoology, University of Ngaoundere. The set-ups were arranged in a completely randomized block and carried out under the laboratory conditions specified above.

### 2.3. Bioassays

#### 2.3.1. Mortality and Progeny Production Inhibition by Weevils Exposed to the Terpenes

Fifty grams of maize grains were placed in 500 mL glass jars. Aliquots of the α-pinene and 3-carene were applied at the following dosages 0 (control), 0.8, 4, 8 and 12 ppm (diluted in 1 mL acetone). Control treatments received 1 mL acetone only. All treatments were replicated 4 times. The maize-monoterpene-acetone mixture was then hand-shaken to permit complete coating of the maize by the terpenes. After hand-shaking, the jars were left open for 45 min on laboratory shelves to permit complete evaporation of the solvent. Thereafter, 20 adult (≤7 d old) *S. zeamais* of mixed sexes were added to each jar and the jars were replaced on laboratory shelves. Insect mortality was recorded 1, 3, 7 and 14 days following treatment and percentage insect mortality was corrected using an previously described formula [20]. Insects were considered dead when no leg or antennal movements were observed when touched with entomological forceps.

On the 14th day post-infestation, the surviving weevils were transferred to new jars containing maize grains and kept under the same experimental conditions. The weevils were allowed 5 weeks to produce new progeny. The recording of F1 adults commenced from the 6th week post-infestation and was continued for the following 5 weeks [21]. The percentage of reduction in adult emergence or inhibition rate (% IR) was calculated with reference to the F1 adult weevils from the untreated control.

#### 2.3.2. Grain Damage

Quantities of 100 g maize grain were measured and placed in 1000 mL glass jars. Aliquots of the α-pinene and 3-carene were applied separately at the following dosages 0 (control), 0.8, 4, 8 and 12 ppm (diluted in 2 mL acetone). Control treatments received 2 mL acetone only. All treatments were replicated 4 times totaling 20 jars for each of the monoterpenes. The maize-monoterpene-acetone mixture was then hand-shaken to permit complete coating of the maize by the terpenes. After shaking the jars, they were left open for 45 min on laboratory shelves to permit complete evaporation of the solvent. Thereafter, unsexed 20 adults (≤7 d old) *S. zeamais* were separately added into each jar and kept on laboratory shelves for 90 days. Grain damage was determined as follows: 100 grains were randomly selected from each jar and the number of damaged (grains with characteristic holes) and undamaged grains were counted.

#### 2.3.3. Fumigant Effect of Monoterpenes on the Maize Weevil

20 unsexed adult weevils (≤7 d old) were placed in 1000 mL glass jars with the mouth screened with filter papers (10 cm diameter) that were held in place with rubber bands. Four different concentrations (1, 2, 3 and 4 ppm) of α-pinene and 3-carene were separately dispensed on each of the filter papers following which the jars were sealed with aluminum foil. Control treatments received no monoterpene. Mortality was evaluated 1, 4, 8, 12, 16, 20 and 24 h after treatment. All treatments were replicated 3 times.

#### 2.3.4. Repellence Test

The repellence test used was adopted from previous studies [21,22]. Four solutions of 1, 2, 3 and 4 µL of monoterpenes were separately added to 4 different 300 mL transparent bottles containing 50 g of maize. Five per cent commercial Poudrox^®^ (a malathion-based locally used insecticide) was used as positive control, while negative control bottles contained only maize. Treatment bottles containing monoterpenes and their corresponding controls in each case were then linked neck-to-neck with a 14 cm (3 cm diameter) plastic tubing. Twenty unsexed weevils (≤7 d old) were introduced through a one-cm (diam) hole made at the midway on the tube linking the pair of jars. The hole was sealed and the set-up was kept in the dark to exclude the influence of partial illumination as the weevils made choices of the bottles. The numbers of the weevils that walked to the bottles treated monoterpenes and control bottles were recorded in diffused light after one hour. Weevils that remained in the tubes were considered not to have made a choice. The percentage repellence (PR) was calculated using the formula **PR = 2 × (C − 50)** where: C is the percentage of insects in the negative control half [23]. The results were interpreted following a scale in previous similar study [22] (Table 1). All treatments were replicated 4 times.

#### 2.3.5. Contact and Fumigant Effect of the Monoterpenes on the Development of *S. zeamais* Immature Stages

Fifty grams of maize grains were placed in 500 mL glass jars. Twenty unsexed weevils (≤7 d) were added to the maize grains. The jars were then closed with perforated lids to permit ventilation and thereafter, the jars were placed on shelves in the laboratory for 14 days to permit the laying of eggs after which the adult weevils were removed. The maize grains containing the developing weevils were used to investigate contact and fumigant effect of the monoterpenes on weevil development.

To investigate contact effect of the monoterpenes on developing weevils varying concentrations of the monoterpenes; 0 (control), 0.8, 4, 8 or 12 mg (dissolved in 1 mL acetone) were added to 1 kg of the infested maize grain. The infested maize and the monoterpene solution were manually shaken and left exposed for 45 min to permit solvent evaporation. Following complete evaporation of the solvent, the jars were sealed with filter paper.

To investigate the effect the monoterpenes as fumigants on the developing stages of the weevil, 0 (control), 0.8, 4, 8 and 12 mg of monoterpenes dissolved in 1 mL of acetone were dispensed on 25 cm (diam) filter papers placed directly under the lids of jars containing 1 kg of infested maize kernels. The jar lids were screwed tight to create hermetically sealed systems.

After treatment, the jars were placed on laboratory shelves and observed weekly from 28th day [21] for F1 adults for 5 weeks. All of the treatments were replicated 4 times.

### 2.4. Data Analysis

Adult mortality in 1, 3, 7 and 14 d post-treatment was corrected relative to natural mortality in the controls using a correction formula [20]. Data was analyzed using SPSS (Statistical Package for Social Sciences) software [21]. Percentage mortality values were arcsine of square-root transformed before analyses to minimize variances and standardize means and all experimental treatments were evaluated for statistical differences using ANOVA statistics, while the Tukey test (HSD) was used for mean separation [21]. The dose–mortality response was analyzed by Probit analysis [24] using the maximum likelihood estimation.

## 3. Results

### 3.1. Mortality of Adult Weevils Resulting from Contact Toxicity of Monoterpenes

Mortality generally increased with concentration of monoterpene and period of insect exposure (Table 2). At all concentrations, mortality of weevils after the first day of treatment ranged between 3% with exposure to 0.08 ppm α-pinene and 92% with exposure to 12 ppm of 3-carene (Table 2). Following 3 d exposure period, 3-carene was more active than α-pinene as 3-carene effected significantly higher mortality than α-pinene (*p* < 0.05). However, the highest concentrations of both monoterpenes caused 98% weevil mortality by the 14th day of exposure. Lethal Concentration (LC_50_) values also decreased progressively with the period of exposure (Table 2). Application of α-pinene resulted in the decreased of LC_50_ from 13.394 ppm on the first day to 4.133 ppm on the 14th day. Weevil mortality was concentration-dependent and exposure-time-dependent for all of the investigated monoterpenes (*p* < 0.0001).

### 3.2. Effect of Contact Toxicity on Weevil Progeny Production

**Figure 1:**
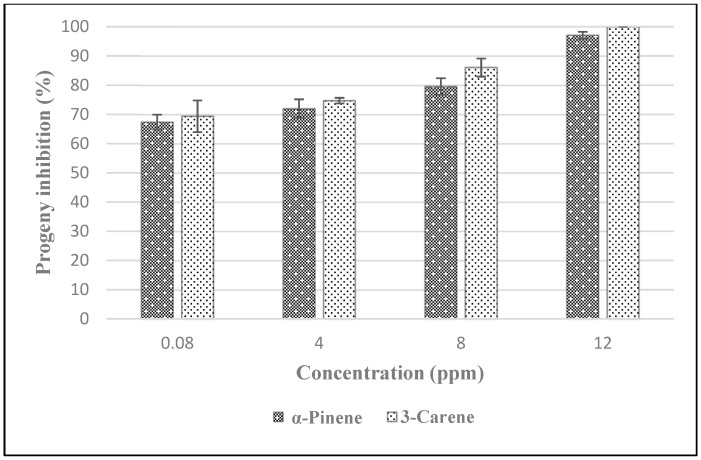
Reduction of *Sitophilus zeamais* progeny production due to treatment of stored maize with α-pinene and 3-carene by contact.

Decrease in progeny production was also dose dependent. At the highest dose (12 ppm), application of α-pinene resulted in progeny reduction of 98% while application of 3-carene caused 100% progeny reduction. Even the application of a low dose (0.08 ppm) of both monoterpenes was effective in reducing progeny production of at least 70% compared to the control (Figure 1). Highly significant differences were observed in the efficacies of the different doses of both compounds, but applications of similar doses of both terpenes did not result in progeny reductions that were significantly different.

### 3.3. Mortality Fumigation

As shown in Table 3, mortality was dose- and exposure-time-dependent as it increased with period of exposure (*p* < 0.0001). Weevil mortalities at similar exposure periods for the two monoterpenes were not significantly different. There were no significant differences between the activities of both monoterpenes at all periods of exposure except after 4 h of exposure. 3-Carene at 3 ppm caused 100% mortality at exposure periods of 12 h or longer. A similar dose of α-pinene (3 ppm) caused 94% mortality only after 24 h of exposure. LC_50_ values decreased significantly with period of exposure (Table 4).

### 3.4. Repellent Effect of the Two Monoterpenes

The most active concentrations (3 and 4 ppm) of both products were within class IV (Repellent class) while concentrations of 1 and 2 ppm were either very lowly repellent (3-carene) or moderately repellent (α-pinene). The positive control, which were malathion-based product (Poudrox^®^), did not have repellent effect on the weevils (Class II, Table 4). There were highly significant differences between the various concentrations of both products, although 3-carene was more active. However, similar concentrations of both monoterpenes showed no significant differences in their activities (Table 4).

### 3.5. Effect of Treatment with Monoterpenes on Weevil Damage of Maize Grains

**Figure 2:**
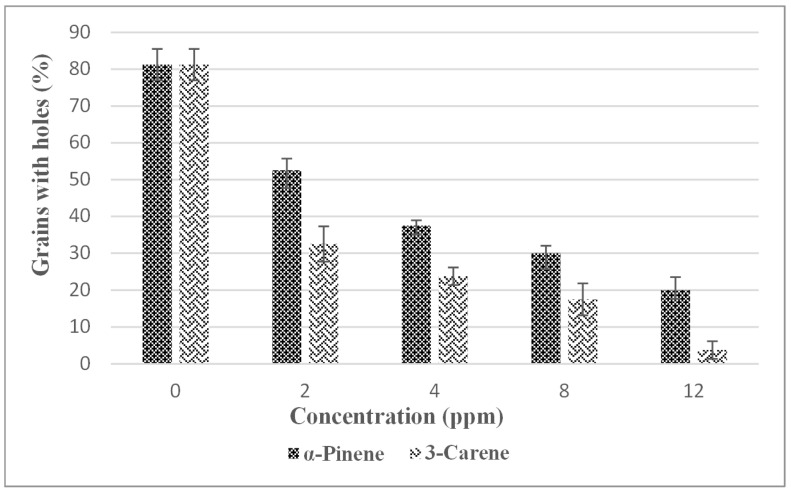
Inhibition of stored maize grain damage by *Sitophilus zeamais* due to treatment with α-pinene and 3-carene.

Damage was evaluated after 3 months of storage. This was evaluated for maize treated by contact application of the monoterpenes. As shown on Figure 2, relative to the untreated control, damage increased with concentration of the monoterpenes. However, 3-carene was more effective in reducing damage compared with α-pinene; at 12 ppm application rate of 3-carene and α-pinene reduced damage after 3 months of treatment by 95% and 77%, respectively. In the untreated control, 85% of the stored maize was damaged after 3 months of storage.

### 3.6. Reduction in the Production of Weevil Progeny by Application of Monoterpenes

For both compounds, efficacy of the monoterpenes in the reduction of progeny production increased with dosage but contact applications of the compounds were more effective than fumigant applications. At the application rate of 12 ppm, α-pinene reduced weevil emergence up to 98% and 92% with contact and fumigant applications, respectively, compared to the control. At similar dose of12 ppm, 3-carene reduced weevil emergence 96% and 95% with contact and fumigation applications, respectively (Table 5).

## 4. Discussion

Monoterpenes just as the essential oils from which they are isolated are very volatile liquids. In the current study, α -pinene and 3-carene were very active especially when used as fumigants (24 h LC_50_ = 1.402 and 0.610 ppm respectively). In a previous study, high activity (LD_50_ = 8.172 μL) of terpenes by fumigation against *Calosobrochus maculatus* and *Sitophilus zeamais* have also been reported [16]. This is mainly due to the ability of these chemicals to penetrate the insect’s respiratory system [25]. These results show however that these two terpenes are more active than other monoterpenoids who after 24 h in single, did not cause up to 100% mortality of *Tribolium castaneum* and *Plodia interpunctella* at 66.7µL/1L [26]. The high activity of α-pinene and 3-carene has been suggested to be due to their ability to penetrate the insects’ respiratory system [26,27]. In this study, 3-carene was more active than α-pinene. The limited insecticidal potentials of α-pinene has also been demonstrated in the larvae of *Tribolium castaneum* Herbst (Coleoptera: Tenebrionidae) [27].

Though not all terpenes from essential oils are bioactive when applied separately, they could work in synergy to increase the bioactivity if applied in combinations or as essential oils. For instance, fenchone that has been demonstrated to be less active than *Pectranthus glandulosus* essential oil from which it was isolated [6,28].

This study demonstrates some monoterpenes could exhibit high toxicity to insect pests even when applied alone. In addition, we demonstrated that contact applications of α-pinene and 3-carene were more active with better LC_50_ = 4.133 and 1.642 ppm, respectively, than the essential oil, *Cupressus sempervirens*, with LC_50_ = 43.06 ppm [7].

The repellent effect of both terpenes on weevils was just around average, while the positive control, a chemical insecticide (Poudrox^®^), did not exhibit any repellent effect on the weevils. A useful pesticide, whether chemical or botanical as is the case with plant extracts, does not necessarily have to be an insect repellent to be effective but for postharvest commodities insect repellency characteristic of any biopesticide may be used to prevent infestation. The repellence of *Cupressus sempervirens,* the essential oil from which these terpenes were extracted has been found to be moderate [29]. Higher repellent effects of plant crude extracts compared to pure Cymol against *S. zeamais* and other storage pests have been reported [7,9]. Furthermore, current results on *S. zeamais* confirmed earlier observation that 3-carene was attractive to 5 different species of forest beetles [30]. Both monoterpenes were not able to generate 100 % mortality of immature weevil stages even when applied at high concentrations.

The pest management strategy that can be deployed against *S. zeamais* using these two monoterpenes should be aimed at the elimination of the adults since they are more susceptible to the monoterpenes compared with the developing stages of the weevil. Eliminating the adults will prevent the laying of eggs that will sustain infestation. In addition, the highly volatile nature of the terpenes could have prevented adult weevils from penetrating the grains to deposit eggs.

## Figures and Tables

**Table 1:** Interpretation of Percentage Repellences.

Class	Repellence Rate (%)	Interpretation
X	>0.01 to <0.1	Non repellent
I	0.1 to 20	Very low repellence
II	20.1 to 40	Low repellence
III	40.1 to 60	Moderately repellent
IV	60.1 to 80	Repellent
V	80.1 to 100	Very repellent

**Table 2:** Cumulative mortality of *Sitophilus zeamais* due to treatment of stored maize with α-pinene and 3-carene by contact.

Period (Days)	Concentration (ppm)	Mortality (%)		*F*_(1,6)_
		α-Pinene	3-Carene	
1	0	0.00 ± 0.00 ^A^	0.00 ± 0.00 ^A^	83.526 ***
	0.08	2.50 ± 1.44 ^A^	3.75 ± 1.25 ^A^	
	4	5.00 ± 2.04 ^A^	8.75 ± 2.39 ^A^	
	8	21.25 ± 2.39 ^B^	65.00 ± 6.12 ^B^	
	12	61.25 ± 2.39 ^C^	90.00 ± 2.04 ^C^	
	*F*_(4,15)_	184.544 ***	173.729 ***	
	LC_50_	13.394	6.348	
3	0	0.00 ± 0.00 ^A^	0.00 ± 0.00 ^A^	25.138 **
	0.08	6.25 ± 2.39 ^A^	11.50 ± 2.47 ^B^	
	4	7.50 ± 1.44 ^A^	14.00 ± 2.27 ^B^	
	8	27.75 ± 3.04 ^B^	87.50 ± 3.23 ^C^	
	12	78.50 ± 3.84^C^	98.75 ± 1.25 ^D^	
	*F*_(4,5)_	163.078 ***	471.845 ***	
	LC_50_	9.720	4.215	
7	0	0.00 ± 0.00 ^A^	0.00 ± 0.00 ^A^	7.118 *
	0.08	11.75 ± 2.30 ^AB^	27.50 ± 3.57 ^B^	
	4	14.50 ± 3.01 ^B^	35.50 ± 2.72 ^B^	
	8	52.00 ± 5.05 ^C^	96.00 ± 2.45 ^C^	
	12	94.50 ± 2.06 ^D^	100.00 ± 0.00 ^C^	
	*F*_(4,5)_	158.057 ***	373.390 ***	
	LC_50_	5.916	2.685	
14	0	0.00 ± 0.00 ^A^	0.00 ± 0.00 ^A^	2.951^ns^
	0.08	20.25 ± 1.11 ^B^	51.50 ± 1.94 ^B^	
	4	27.00 ± 3.20 ^B^	57.00 ± 1.68 ^B^	
	8	67.50 ± 3.80 ^C^	98.50 ± 1.50 ^C^	
	12	97.25 ± 1.60 ^D^	100.00 ± 0.00 ^C^	
	*F*_(4,5)_	271.545 ***	955.712 ***	
	LC_50_	4.133	1.642	

Means ± S.E. in the same column for the same category of insecticide, followed by the same letter do not differ significantly at *p* = 0.05 (Tukey test). Each datum represents the mean of four replicates of 20 insects each. ^ns^: non-significant, *: significant (*p* < 0.01); **: very significant (*p* < 0.001); ***: very highly significant.

**Table 3:** Mortality of *Sitophilus zeamais* due to exposure to α-pinene and 3-carene fumes.

Period (h)	Concentration (ppm)	Mortality (%)		*F*_(1,28)_
		α-Pinene	3-Carene	
1	0	0.00 ± 0.00 ^A^	0.00 ± 0.00	0.135 ^ns^
	1	0.00 ± 0.00 ^A^	0.00 ± 0.00	
	2	0.00 ± 0.00 ^A^	0.00 ± 0.00	
	3	53.33 ± 1.67 ^AB^	5.00 ± 0.00	
	4	5.00 ± 0.00 ^B^	5.00 ± 0.00	
	*F*_(4,10)_	10.00 **	//	
	LC_50_	11.552	11.563	
4	0	0.00 ± 0.00 ^A^	0.00 ± 0.00 ^A^	4.706 *
	1	11.67 ± 1.67 ^B^	5.00 ± 0.00 ^A^	
	2	11.67 ± 1.67 ^B^	13.33 ± 1.67 ^B^	
	3	31.67 ± 1.67 ^C^	13.33 ± 1.67 ^B^	
	4	**61.67 ± 4.41 ^D^**	**18.33 ± 1.67 ^B^**	
	*F*_(4,10)_	106.000 ***	32.500 ***	
	LC_50_	**3.554**	**5.810**	
8	0	0.00 ± 0.00 ^A^	0.00 ± 0.00 ^A^	0.007 ^ns^
	1	16.67 ± 1.67 ^B^	11.67 ± 1.67 ^B^	
	2	21.67 ± 1.67 ^B^	21.67 ± 1.67 ^C^	
	3	46.67 ± 1.67 ^C^	61.67 ± 1.67 ^D^	
	4	96.67 ± 1.67 ^D^	81.67 ± 3.33 ^E^	
	*F*_(4,10)_	637.750 ***	310.857 ***	
	LC_50_	2.466	2.568	
12	0	0.00 ± 0.00 ^A^	0.00 ± 0.00 ^A^	1.519 ^ns^
	1	21.67 ± 1.67 ^B^	33.33 ± 1.67 ^B^	
	2	28.33 ± 1.67 ^C^	48.33 ± 1.67 ^C^	
	3	46.67 ± 1.67 ^D^	100 ± 0.00 ^D^	
	4	100 ± 0.00 ^E^	100 ± 0.00 ^D^	
	*F*_(4,10)_	857.167 ***	1705.500 ***	
	LC_50_	2.265	1.355	
16	0	0.00 ± 0.00 ^A^	0.00 ± 0.00 ^A^	1.783 ^ns^
	1	23.33 ± 1.67 ^B^	55.00 ± 2.89 ^B^	
	2	41.67 ± 4.41 ^C^	61.67 ± 1.67 ^B^	
	3	61.67 ± 6.01 ^D^	100.00 ± 0.00 ^C^	
	4	100.00 ± 0.00 ^E^	100.00 ± 0.00 ^C^	
	*F*_(4,10)_	124.452 ***	761.875 ***	
	LC_50_	1.931	1.062	
20	0	0.00 ± 0.00 ^A^	0.00 ± 0.00 ^A^	2.035 ^ns^
	1	30.00 ± 2.89 ^B^	71.67 ± 4.41 ^B^	
	2	48.33 ± 1.67 ^C^	85.00 ± 2.89 ^C^	
	3	80.00 ± 2.89 ^D^	100.00 ± 0.00 ^D^	
	4	100.00 ± 0.00 ^E^	100.00 ± 0.00 ^D^	
	*F*_(4,10)_	404.286 ***	311.350 ***	
	LC_50_	1.608	0.727	
24	0	0.00 ± 0.00 ^A^	0.00 ± 0.00 ^A^	1.604 ^ns^
	1	31.67 ± 3.33 ^B^	80.00 ± 2.89 ^B^	
	2	56.67 ± 1.68 ^C^	90.00 ± 2.89 ^C^	
	3	91.67 ± 3.33 ^D^	100.00 ± 0.00 ^D^	
	4	100.00 ± 0.00 ^D^	100.00 ± 0.00 ^D^	
	*F*_(4,10)_	346.833 ***	534.000 ***	
	LC_50_	1.402	0.610	

Means ± S.E. in the same column for the same category of insecticide, followed by the same letter, do not differ significantly at *p* = 0.05 (Tukey test). Each datum represents the mean of four replicates of 20 insects each. ^ns^ non-significant, *: significant (*p* < 0.01); **: very significant (*p* < 0.001); ***: very highly significant (*p* < 0.0001).

**Table 4:** Repellence of *Sitophilus zeamais* due to treatment of stored maize with α-pinene, 3-carene and Poudrox^®^.

Product	Concentration (ppm)	Repellence (%)	Class	Interpretation
α-Pinene	Control (5%)	24.31 ± 9.17 ^A^	II	Low repellence
	1	41.84 ± 4.48 ^AB^	III	Moderately repellent
	2	57.43 ± 7.09 ^B^	IV	Repellent
	3	57.62 ± 3.01 ^B^	IV	Repellent
	4	68.04 ± 5.27 ^B^	IV	Repellent
	*F*_(4,15)_	7.617 **		
3-Carene	Control (5%)	24.31 ± 9.17 ^B^	II	Low repellence
	1	4.41 ± 5.18 ^A^	I	Very low repellence
	2	35.00 ± 6.66 ^B^	III	Moderately repellent
	3	60.00 ± 7.07 ^C^	IV	Repellent
	4	66.39 ± 5.45 ^C^	**IV**	Repellent
	*F*_(4,15)_	27.481 ***		

Means ± S.E. in the same column for the same category of insecticide, followed by the same letter do not differ significantly at *p* = 0.05 (Tukey test). Each datum represents the mean of four replicates of 20 insects each. **: very significant (*p* < 0.001); ***: very highly significant (*p* < 0.0001); Control (5%): Poudrox.

**Table 5:** Inhibition of development of *Sitophilus zeamais* immature stages due to treatment of stored maize with α-pinene and 3-carene.

Product	Concentration (ppm)	Proportion of Inhibition (%)		*F*_(1,6)_
		By Contact	By Fumigation	
α-Pinene	**0**	0.00 ± 0.00 ^A^	0.00 ± 0.00 ^A^	**//**
	2	38.48 ± 2.53 ^B^	32.98 ± 7.30 ^B^	**0.507 ^ns^**
	4	70.44 ± 6.32 ^C^	57.41 ± 5.12 ^C^	**2.567 ^ns^**
	8	86.59 ± 3.55 ^C^	71.28 ± 2.99 ^C^	10.896 *
	12	97.60 ± 1.40 ^D^	91.29 ± 1.68 ^D^	8.332 *
	***F*_(4,15)_**	128.995 ***	68.551 ***	
3-Carene	**0**	0.00 ± 0.00 ^A^	0.00 ± 0.00 ^A^	//
	**2**	44.67 ± 4.53 ^B^	42.11 ± 6.25 ^B^	**0.110 ^ns^**
	**4**	56.10 ± 2.16 ^B^	66.07 ± 9.77 ^BC^	**0.991 ^ns^**
	**8**	85.66 ± 3.70 ^C^	76.47 ± 4.75 ^CD^	**2.330 ^ns^**
	**12**	95.26 ± 1.79 ^C^	94.65 ± 1.95 ^D^	**0.826 ^ns^**
	***F*_(4,15)_**	168.719 ***	41.487 ***	

Means ± S.E. in the same column for the same category of insecticide, followed by the same letter do not differ significantly at *p* = 0.05 (Tukey test). Each datum represents the mean of four replicates of 20 insects each. ^ns^ non-significant, *: significant (*p* < 0.01); ***: very highly significant (*p* < 0.0001).
